# Death from Chloral

**Published:** 1874-07

**Authors:** 


### DEATH FROM CHLORAL.

As an illustration of what pretentious ignorance will do. A few weeks ago, in the central part of this state, (Ohio,) near Lancaster, a man by the name of Phillips claiming to be a dentist, administered to a young lady, Miss. Emma Jones, 80 grains of Hydrate Chloral from which she died in a few hours, the man immediately left for parts unknown.

This man should have been arrested and punished for manslaughter, at least. And yet there are perhaps an hundred practicing dentistry in the State of Ohio no better qualified than he; perhaps not all just as rash, but possibly some of them more so. All of them, through ignorance, if not culpable rashness, are liable to do incalculable and irremediable mischief.

How long must these things be?

Who is safe in such hands?

And yet some people insist that free license and full scope be given to every charlatan or scoundral who may assume the title of physician or dentist to ply his death dealing trade *ad libitum.*

